# Supplementing the Diet of Dairy Goats with Dried Orange Pulp throughout Lactation: II Effect on Milk Fatty Acids Profile, Phenolic Compounds, Fat-Soluble Vitamins and Antioxidant Capacity

**DOI:** 10.3390/ani11082421

**Published:** 2021-08-17

**Authors:** Manuel Delgado-Pertíñez, Ignacio Martín-García, Yolanda Mena, Luis Ángel Zarazaga, José Luis Guzmán

**Affiliations:** 1 Departamento de Agronomía, Escuela Técnica Superior de Ingeniería Agronómica, Universidad de Sevilla, Ctra. Utrera km 1, 41013 Sevilla, Spain; pertinez@us.es (M.D.-P.); yomena@us.es (Y.M.); 2 Estación Experimental del Zaidín (CSIC), Profesor Albareda 1, 18008 Granada, Spain; ignacio.martin@eez.csic.es; 3 Departamento de Ciencias Agroforestales, Escuela Técnica Superior de Ingeniería, Universidad de Huelva, “Campus de Excelencia Internacional Agroalimentario, ceiA3”, Campus Universitario de la Rábida, Carretera Huelva-Palos de la Frontera s/n, 21819 Palos de la Frontera, Spain; zarazaga@uhu.es

**Keywords:** alternative feedstuffs, orange by-product, Payoya breed, fatty acid profile, phenolic compounds, fat-soluble vitamins, full lactation

## Abstract

**Simple Summary:**

Spain is a major global producer of both goats and oranges. The orange juice industry produces high levels of organic waste that could be used as alternative feedstock for ruminants, enhancing dairy farm sustainability by lowering feed costs and reducing the environmental impact. An example of such organic waste is dried orange pulp (DOP), which has been proven beneficial as a 40% or 80% replacement for cereal in the diet of goats at an early lactation stage; therefore, it is pertinent to study these by-products over a longer period, such as the complete lactation cycle of 180 days. This study evaluated the Payoya dairy breed and the resultant milk’s antioxidant composition and fatty acid (FA) content in terms of saturated, monounsaturated, and polyunsaturated FAs (SFA, MUFA, and PUFA, respectively). The levels of vitamin E, total phenolic compounds, and antioxidant capacity in milk increased as the percentage of DOP replacing cereals increased. Moreover, the inclusion of DOP improved the nutritional value of the milk for human health (according to the thrombogenicity index, MUFA/SFA, and PUFA/SFA ratios), especially at the end of lactation, leading to the conclusion that DOP might be an appropriate alternative to cereals in the diets of goats.

**Abstract:**

Although dried orange pulp (DOP) as a short-term dietary supplementation has been proven an effective substitute for cereals in goat diets–without impairing milk quality–there have been no studies considering its use over the full lactation period. This study evaluated replacing cereal with DOP in goat diets for the full 180-day lactation period on milk’s fatty acid (FA) and antioxidant composition. Payoya goats were assigned to three diet groups: a control group consuming a commercial concentrate with alfalfa hay as forage; a DOP40 or DOP80 group, wherein 40% or 80% of the cereal in the concentrate was replaced by DOP. The α-tocopherol and phenolic compounds levels and the antioxidant capacity in the milk increased as the DOP percentage increased. Including DOP might improve the FA indices of milk in the context of human health, especially when included at the end of lactation because it contributes to reducing the thrombogenicity index and increasing both the monounsaturated/saturated FA and polyunsaturated/saturated FA indices and the amounts of indispensables α-C18:3 n-3 and C18:2 n-6 *cis*. Ultimately, DOP presents a plausible alternative to cereals in the diet of goats throughout lactation to improve the nutritional milk quality, especially the healthy antioxidant capacity.

## 1. Introduction

The role of the human diet in reducing the progression of chronic disease has become increasingly important [1], with a diet’s fatty acid (FA) profile being considered an important health determinant [2]. Saturated FA (SFA) intake is generally recognized as harmful, while higher consumption of monounsaturated FA (MUFA) and polyunsaturated FA (PUFA) has been reported as associated with a reduced risk of cardiovascular disease (CVD) [3]. Hence, the Mediterranean Diet Score (MDS) features the MUFA/SFA ratio in its index, and the Alternative Healthy Eating Index (AHEI) features the PUFA/SFA ratio [1]. Linoleic acid (C18:2) and alfa-linolenic acid (α-C18:3 n-3) have been demonstrated to be indispensable because they cannot be synthesized by humans, and acceptable intake has been defined for both types of FA [1]. Additionally, a relationship between human health and the n-6/n-3 ratio has been reported, with a value of less than four proposed to reduce the risk of CVD [4]. Ulbricht and Southgate [5] proposed two indices which might better characterize the diet’s atherogenic (refers to the FA aggregation forming a plaque in the arteries) and thrombogenic (refers to the tendency to form clots in the blood vessels) potential. The type of dietary fat consumed might contribute to both of these processes; C14:0 and C16:0 have great roles both in atherogenesis and thrombogenesis, while C12:0 has a greater role in atherogenesis and C18:0 has a greater role in thrombogenesis. Minor FAs, such as conjugated linoleic acid (CLA), have been reported to have anticancer effects [6] or protect against CVD [7]. Finally, foods containing natural antioxidants (such as vitamins A, C and E, carotenoids, phenolic compounds) have become popular because antioxidants can neutralize free radicals and their deleterious effects in both humans and animals, as well as negating the oxidation processes which can result in a deterioration of nutritional quality [8,9].

Recent research has demonstrated the benefits of full-fat dairy consumption [10]; in general, evidence suggests that milk has a neutral effect on CVD. Furthermore, milk and other dairy products contain natural antioxidants, such as fat-soluble vitamins (such as A and E), carotenoids, sulfur (containing amino acids), enzyme systems, oligosaccharides, peptides, phosphate, zinc, and selenium [11]. Milk might also contain appreciable amounts of phenolic compounds [12,13], which present antioxidant activity too [12]. In general, the content of FAs and the bioactive molecules in milk change in quantity and quality mainly according to diet-related factors [14,15].

The use of local resources, especially when they are recovered from by-products, might significantly enhance dairy farm sustainability. Organic waste from the orange juice industry in Spain is substantial, given that Spain is the leading orange producer in Europe (3.9 million tonnes in 2018) [16]. An example of such organic waste is dried orange pulp (DOP), a by-product that represents up to 15% *w*/*w* of the input. In the context of animal feed, it can be used as a source of both energy and, importantly, bioactive compounds, including phenolic compounds and vitamin E [17,18,19], compounds that have health-related properties, especially based on their antioxidant activity [8,9]. However, few studies have considered the substitution of cereals with DOP in goat diets or the effect this might have on the resultant milk’s FA and antioxidant properties [20,21]. In this experimental study’s previous paper [21], which evaluated the effects of replacing cereal with DOP in the diet of goats during the early lactation phase, diet had no significant effect on FA content. However, the resultant milk’s α-tocopherol, total phenolic compound (TPC), and total antioxidant capacity (TAC) increased. Accordingly, this study was designed to evaluate the effects of replacing cereal with DOP in the diet of dairy goats for the complete 180-day lactation on the FA and antioxidant composition of their milk.

## 2. Materials and Methods 

### 2.1. Experimental Diets, Goats, and Experimental Procedure

The experiment was performed with lactating Payoya goats at the University of Huelva’s (Huelva, Spain) experimental farm over the duration of their 180-day lactation. Animals were handled in accordance with the Spanish guidelines for experimental animal protection (Royal Decree 53/2013).

DOP pellets were prepared using orange juice residues, following a conventional industrial process (Cítricos del Andévalo, S.A., Huelva, Spain) as described by Guzmán et al. [22].

As previously described [21], forty-four primiparous goats were assigned to three diet treatments, with each group housed in a communal pen: the control (CD, *n* = 14) was given commercial concentrate plus alfalfa hay as forage; the DOP40 (*n* = 16) group was based on the CD but with 40% of the cereals in the concentrate replaced with DOP; finally, the DOP80 (*n* = 14) group was based on the CD but with 80% of the cereals in the concentrate replaced with DOP. The groups were balanced according to the live weight (LW) (37.0 ± 1.26, 38.0 ± 1.33 and 37.5 ± 2.12 kg for the Control, DOP40, and DOP80 groups, respectively) and body condition scores (BCS) (2.76 ± 0.09, 2.51 ± 0.06, and 2.55 ± 0.08 for the Control, DOP40 and DOP80 groups, respectively) of the goats. After delivery, the animals were fed the experimental diet adapted for early-to-mid stages of lactation; approaching the late stage, over 300 g of concentrate were removed from the diet. The ingredients and the nutritive value of the rations are shown in Table 1; they were designed (isoenergetic and isoproteic) using the Feed Ration Balancer (Format Solutions) software, version 2.0 (2017; Cargill, Inc., Wayzata, MN, USA).

The goats were fed once daily—in the mornings—and food intake for each group was calculated daily by subtracting the orts from the amount of food offered every day. For forage, the average daily consumption per animal was 0.4 kg; for concentrate, the average daily consumption per animal was either 1.88 kg (early lactation, up to 60 d, see Guzmán et al. [21]) or 1.81 kg (mid-lactation, up to 120 d) or 1.57 kg (late lactation, up to 180 d). After weaning the kids at 28–32 days old, the average total intake per goat was calculated for each group for early (for CD, DOP40 and DOP80, respectively: DM was 2.04, 2.00 and 1.98 kg/d; crude protein was 0.36, 0.33 and 0.36 kg/d; gross energy was 9.28, 8.90 and 8.74 Mcal/d), mid (for CD, DOP40 and DOP80, respectively: DM was 1.92, 1.92 and 1.89 kg/d; crude protein was 0.34, 0.32 and 0.35 kg/d; gross energy was 8.76, 8.53 and 8.35 Mcal/d) and late lactation (for CD, DOP40 and DOP80, respectively: DM was 1.78, 1.76 and 1.75 kg/d, crude protein was 0.32, 0.29 and 0.32 kg/d, and gross energy was 8.14, 7.83 and 7.75 Mcal/d).

After weaning the kids, the animals began being milked once a day (at 09:00 a.m.) in a 12-stall Casse system milking parlor. Coinciding with the test-day milk yield recordings, representative samples from each animal (50 mL aliquots placed in plastic bottles) were taken from the volumetric flask at early lactation (55 ± 5 [21]), mid-lactation (120 ± 5), and late lactation (180 ± 5) days post-partum. The aliquots were frozen at −20 °C until analysis, except for the samples for vitamin analysis, which were frozen at −80 °C.

### 2.2. Feed and Milk Chemical Analyses

A sample of diets (hay and concentrates) at each lactation stage was prepared for analysis by mixing equal amounts of subsamples collected throughout the lactation and storing them at 4 °C. Before analysis, the samples were dried and ground using a Wiley mill with a 1-mm screen. This sampling approach was previously described by Guzmán et al. [21], with AOAC [23] methods being used to determine the dry matter, ash, N content, and ether extract; the analyses of neutral detergent fiber (NDF), acid detergent fiber (ADF) and acid detergent lignin (ADL) were conducted according to Van Soest et al. [24], the gross energy (GE) content being determined using an adiabatic calorimeter, according to the manufacturer’s instructions, the forage unit for lactation (UFL) and protein digestible in the small intestine (PDI) being calculated using the Feed Ration Balancer software, the TAC being analyzed by the DPPH (2,2-diphenyl-l-picrylhydrazyl) assay as described by Shin et al. [25], the TPC content being estimated according to the procedure described by Seiquer et al. [26], with some modifications, and, finally, the methods described by Delgado-Pertíñez et al. [27] and Gutiérrez-Peña et al. [28] being used to determine the FA profile and vitamin E (α-tocopherol) content. The details for the analyses of TAC, TPC, FA, and vitamin E in feed samples can be found in the work of Guzmán et al. [21].

For the milk samples—as previously described by Guzmán et al. [21]—fat content was estimated using near-infrared spectroscopy (NIR). Fat extraction of milk and the direct methylation of FAs were performed in a single-step method developed by Sukhija and Palmquist [29] and revised by Juárez et al. [30] according to the work of Delgado-Pertíñez et al. [27] and Gutiérrez-Peña et al. [28]. Separation and quantification of FA methyl esters (FAMEs) were carried out using a gas chromatograph (Agilent 6890N Network GS System, Agilent, Santa Clara, CA, USA) equipped with a flame ionization detector (FID) and automatic sample injector HP 7683, and fitted with an HP-88 J&W fused silica capillary column (100 m, 0.25 mm i.d., 0.2-µm film thickness; Agilent Technologies Spain, S.L., Madrid, Spain). Nonanoic acid methyl ester (C9:0 ME) was used as an internal standard (Sigma Aldrich Co., Madrid, Spain). Individual FAs were identified by comparing their retention times with those of the authenticated standard FA mix Supelco 37 (Sigma, Madrid, Spain). The CLA (conjugated linoleic acid) isomers (*cis*9, *trans*11, and *trans*10, *cis*12) were identified by comparing retention times with those of another authenticated standard (Matreya, LLC, Pleasant Gap, PA, USA). Fat-soluble vitamin (A and E) measurement of milk was based on procedures developed Herrero-Barbudo et al. [31] and Chauveau-Duriot et al. [32] and modified by Gutiérrez-Peña et al. [28]. A chromatographic analysis was carried out on an Acquity UPLC, with a fluorometric detector, isocratic pump, PDA, and 150 × 2.1 mm Acquity UPLC HSS T3 1.8-µm column (Waters, Saint-Quentin-en-Yvelines, France). Tocopherols and retinol were positively identified by comparing their retention times with those of high purity standards of the measured substances (all-*trans*-retinol, α-tocopherol, β-tocopherol, and γ-tocopherol; Sigma, Madrid, Spain). Other standards of high purity (retinyl acetate, retinyl palmitate, and tocopheryl acetate; Sigma) were used as internal standards. TAC was determined using the ABTS (2,2′-azino-bis [3-ethylbenzothiazoline-6-sulphonic acid]) procedure developed by Fellegrini et al. [33] and modified by Delgado-Pertíñez et al. [27]. The milk sample was added to 1 mL of ABTS solution and incubated at 25 °C for 10 min. Scavenging of the ABTS+ radical was monitored by the absorbance decrease at 730 nm. The water-soluble vitamin E analog Trolox was used as a standard, and TAC was expressed as mmol Trolox equivalents. Finally, TPC was analyzed according to the method described by Vázquez et al. [34] as modified by Guzmán et al. [21]. The phenolic compounds in the liquid extract were quantified at an absorbance of 750 nm using the Folin–Ciocalteu method, adapted to test tubes. Standard solutions of gallic acid (GA) were used to express the phenolic compounds as g of GA equivalents.

### 2.3. Data Treatment and Statistical Analysis

The data for the milk composition of the goats was recorded at each lactation stage and analyzed according to the repeated measures procedure using IBM SPSS Statistics for Windows (version 26.0; IBM Corp., Armonk, NY, USA). The model included the fixed between-subjects factors of dietary treatment (CD, DOP40, or DOP80) and prolificacy (single or double birth) and the fixed within-subjects factor of lactation stage (repeated measures), as well as the interactions between these factors. For simplification, the results for the factor prolificacy have not been presented in this paper. Tukey’s Honest Significant Difference (HSD) test was used for pairwise means comparisons. Finally, Pearson correlation coefficients were calculated for some of the variables used.

## 3. Results

### 3.1. Fat-Soluble Vitamins, Phenolic Compounds, and Antioxidant Capacity

Average milk fat yield and percentage were only affected by the lactation phase (Table 2). The percentage increase in fat between the early and late stages produced the highest values for the mid and late stages and the lowest values for the early stage; the opposite pattern was observed for fat yield, with the highest value obtained at the early stage and the lowest values being detected at the mid and late stages.

**Figure 1:**
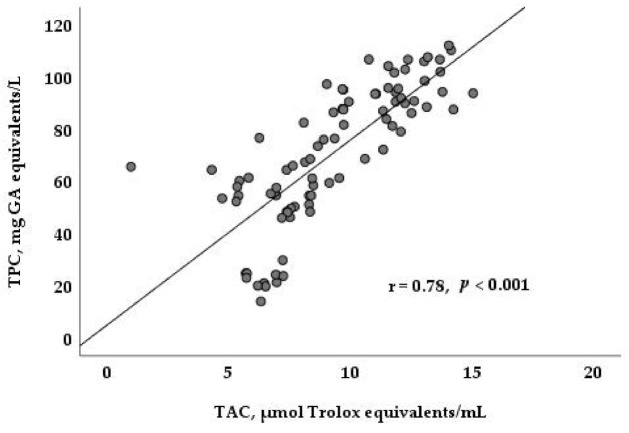
Correlation between total antioxidant capacity (TAC) and total phenolic compounds (TPC) in milk derived from goats fed different diets and at different lactation stages.

The average content of all antioxidant parameters changed significantly between diets and lactation phases (Table 2), with the exception of retinol content, which was not affected by diet, although it did increase from the early to the late lactation phase (*p* < 0.01). The milk fat of goats fed the DOP80 diet presented higher values of α-tocopherol (45.2 μg/100 g) than the other diets (21.1 and 28.2 μg/100 for the CD and DOP40 diets) (*p* < 0.01). This vitamin was also affected by the lactation phase (*p* < 0.01), decreasing between the early and mid-phases and increasing thereafter. Significant effects of the main factors and interaction between them were observed in the content of TPC and TAC. The TPC and TAC content were higher for the DOP80 diet (97.0 mg GA equivalents/l; 12.0 μmol Trolox equivalents/mL) than for the CD diet (46.3 mg GA equivalents/l; 6.44 μmol Trolox equivalents/mL), while the DOP40 diet (69.1 mg GA equivalents/l; 9.39 μmol Trolox equivalents/mL) presented an intermediate value between the other two (*p* < 0.001). Regarding the interaction between diet and lactation stage, the content of both parameters decreased (*p* < 0.001) from the early to the late lactation phase, with the exception of TPC for the DOP80 diet, which did not decrease during the late phase, and the TAC in the DC diet, which did not present any between-phase differences (*p* < 0.05). Finally, Figure 1 demonstrates the positive correlation between TAC and TPC (r = 0.78, *p* < 0.001) for all three diets and throughout lactation.

### 3.2. Fatty Acid Composition

Significant effects of the main factors and interactions between them were observed for most individual FAs, FA groups, and FA indices, with differences between diets especially notable in the late stage (Table 3). Effects of the main factors were observed in the C10:0, C11:0, C12:0, and C20:0 content. Effects for only the lactation phase were observed in the content of C4:0, C13:0, C22:0, C22:2, C24:0, and total n-3, as well as impacting the AI index. Finally, no effects were observed in C14:1, C15:1, C17:0, C20:1 n9, C20:2, C20:3 n3, C20:5 n-3 (EPA), C21, C22:1 n9, C22:6 n-3 (DHA), C23:0 or C24:1 content.

**Figure 2:**
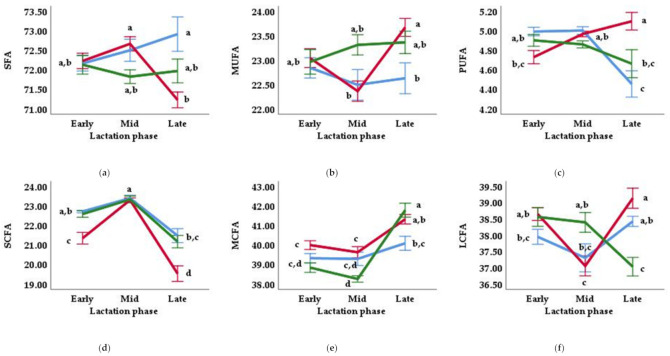
Interactive effect of the dietary treatment and the lactation phase (early, mid, late) on different types of fatty acids (FAs): saturated FAs, SFA (**a**); monounsaturated FAs, MUFA (**b**); polyunsaturated FAs, PUFA (**c**); short-chain FAs, SCFA (**d**); medium-chain FAs, MCFA (**e**) and long-chain FAs, LCFA (**f**). The three diets were the control (blue line), DOP40 (red line), and DOP80 (green line). Values presented are the means (g/100 g FA), with ^a,b,c,d^ indicating differences between mean values (*p* ≤ 0.05).

With the exception of C4:0 content, which was not affected by diet, total and individual short-chain FAs (SCFA) in milk fat were significantly affected by this factor (*p* < 0.001; although the milk fat of goats fed the CD diet presented higher values than the DOP diets, the DOP80 diet did not differ significantly from the CD diet) and by the lactation phase (*p* < 0.001; content was generally lower in late lactation compared to early and mid-lactation) (Table 3). Additionally, C6:0 (*p* < 0.01), C8:0 (*p* < 0.05) and total SCFA (*p* < 0.01) demonstrated interaction between the two main factors (Table 3 and Table 4 and Figure 2); thus, no significant differences were found between the diets during mid-lactation.

Significant effects of the main factors and interaction between them were observed in the C14:0, C16:0, and total medium-chain FAs (MCFA) of milk fat (Table 3 and Table 4 and Figure 2). These effects were generally noted in early and mid rather than late lactation; in particular, the C16:0 content presented the most substantial difference between the diet treatments (generally, the milk fat from goats fed the DOP80 diet produced lower values than the other two groups). Opposite patterns were observed for the other MCFA, which was generally lower in the DOP diets compared to the CD diet and lower in the late stage compared to the early and mid-stages (Table 3). Interaction between the main factors was observed for C15:0 (*p* < 0.001), C16:1 (*p* < 0.01) and DI C16:0 (*p* < 0.01) (Table 3 and Table 4)—only significant differences were found between the diets in the late phase—with the lowest values being obtained in the milk fat of goats fed the DOP40 and DOP80 diets (for C15:0, 0.45 and 0.46 g/100 g FA; for C16:1, 0.96 and 0.97 g/100 g FA; for DI C16:0, 0.04 and 0.04) compared to the CD diet (for C15:0, 0.55 g/100 g FA; for C16:1, 1.08 g/100 g FA; for DI C16:0, 0.05).

The total and individual long-chain FAs (LCFA) content in the milk fat was mainly affected by the lactation phase—generally decreasing from the early to mid-phase and increasing thereafter—and by the interaction between diet and lactation phase (Table 3 and Table 4 and Figure 2). Diet mainly affected the octadecenoic acids of milk fat; specifically, the C18:1 n-9 *cis* content was higher for the DOP80 than the CD group, while the DOP40 did not differ from the other two groups (*p* < 0.05). Regarding the interaction between diet and lactation stage, significant differences were generally detected between diets in the late stage (Table 4). The C18:0 content was lower for the DOP80 diet (10.0 g/100 g FA) than the CD diet (12.4 g/100 g FA), while the DOP40 diet (11.4 g/100 g FA) presented an intermediate value between the other two (*p* < 0.001). Regarding unsaturated FAs, the two DOP diets showed greater values for the FAs C18:1 n-11 *trans* (*p* < 0.01), C18:1 n-9 *cis* (*p* < 0.05), C18:2 n-6 *trans* (*p* < 0.001), C18:2 n-6 *cis* (*p* < 0.001), α -C18:3 n-3 (*p* < 0.01) and CLA *trans*-10, *cis*-12 (*p* < 0.001). However, the CD diet, compared with the DOP diets, showed greater values for the FAs C18:1 n-9 *trans* (*p* < 0.01), γ-C18:3 n-6 (*p* < 0.001), CLA *cis*-9, *trans*-11 (*p* < 0.01), C20:3 n-6 (*p* < 0.001) and C22:5 n-3 (*p* < 0.001). The DI C18:0 was affected by diet (*p* < 0.01) and interaction (*p* < 0.001) between the main factors (Table 3 and Table 4), meaning significant differences between diets were only detected during late lactation, with the greatest value being obtained by the DOP80 diet (0.69), the lowest value being observed for the CD diet (0.63), and an intermediate value being observed for DOP40 (0.66).

The total SFA in the milk fat was affected by the diet treatment (*p* < 0.05) and by the interaction between diet and lactation phase (*p* < 0.001) (Table 3 and Figure 2). The main differences between diets were identified during late lactation; that is, the milk fat of goats fed the CD diet presented higher values (72.9 g/100 g FA) than those fed the DOP40 and DOP80 diets (71.2 and 72.0 g/100 g FA), although the DOP80 diet did not differ significantly from the CD diet. Total MUFA was affected by the two main factors (*p* < 0.05) and by the interaction between them (*p* < 0.05) (Table 3 and Figure 2), which decreased between early and mid-lactation and increased thereafter, with the main differences between the diets (content was generally higher in the DOP diets than the CD diet) being identified in the mid (22.5 (CD), 22.4 (DOP40) and 23.3 (DOP80) g/100 g FA) and late (22.6 (CD), 23.7 (DOP40) and 23.4 (DOP80) g/100 g FA) lactation phases. Consequently, significant differences were observed in the MUFA/SFA ratio, with the values being affected by both diet (*p* < 0.05) and the interaction between diet and lactation phase (*p* < 0.01) (Table 3 and Table 4), decreasing between early and mid-lactation and increasing thereafter, with the main differences between the diets (the values generally being higher for the DOP diets compared to the CD diet) being identified in the mid (0.31 (CD), 0.31 (DOP40) and 0.32 (DOP80)) and late (0.31 (CD), 0.33 (DOP40) and 0.33 (DOP80)) lactation phases. The total PUFA content was affected by the lactation stage (*p* < 0.05) and by the interaction between diet and lactation stage (*p* < 0.001) (Table 3 and Figure 2); that is, significant differences between diets were only detected during the late lactation phase, with the greatest value being obtained by the DOP40 diet (5.10 g/100 g FA), the lowest value obtained by the CD diet (4.45 g/100 g FA), and an intermediate value observed for DOP80 (4.66 g/100 g FA). Consequently, significant differences were observed in the PUFA/SFA ratio between diets, but only during late lactation (*p* < 0.001; Table 3 and Table 4), and a higher value was obtained by the milk fat of the DOP40 and DOP80 goats (both 0.07 compared to 0.06 for CD goats). The AI index was only affected by the lactation stage (*p* < 0.001) (Table 3), with a higher value being obtained in the milk fat derived from late lactation (2.27) compared to early and mid (2.10) lactation.

Total n-3 PUFA content in the milk fat was only affected by the lactation stage (*p* < 0.001, Table 3), with the greatest value being derived from late lactation (0.36 g/100 g FA), the lowest value derived from early lactation (0.33 g/100 g FA), and an intermediate value being derived during mid-lactation (0.35 g/100 g FA). Total n-6 PUFA content and n-6/n-3 ratio were affected by both lactation stage (*p* < 0.05 and *p* < 0.001 for n-6 and n-6/n-3 ratio, respectively) and the interaction between diet and lactation stage (*p* < 0.001 and *p* < 0.01 for n-6 and n-6/n-3 ratio, respectively) (Table 3 and Table 4). Notably, significant differences were only detected between diets during late lactation, with the greatest values being obtained for the DOP40 diet (for n-6, 4.01 g/100 g FA; for n-6/n-3, 11.3), the lowest value being obtained for the CD diet (for n-6, 3.28 g/100 g FA; for n-6/n-3, 9.41) and an intermediate value being observed for DOP80 (for n-6, 3.64 g/100 g FA; for n-6/n-3, 10.2). Total CLA content was affected by the two main factors (*p* < 0.05) and by the interaction between them (*p* < 0.001) (Table 3 and Table 4), with significant differences only being found between diets in the late phase. The lowest values were derived from the milk fat of goats fed the DOP40 and DOP80 diets (0.65 and 0.59 g/100 g FA compared to 0.76 g/100 g FA for the CD diet). Similarly, the CLA index was affected by both the lactation stage (*p* < 0.05) and by the interaction between the main factors (*p* < 0.001) (Table 3 and Table 4); that is, significant differences were found between diets in the late phase, with the lowest values being derived from the milk fat of the DOP40 and DOP80 diets (0.46 and 0.45 compared 0.53 for the CD diet). Finally, the TI index was affected by diet (*p* < 0.05), with the lowest value observed for the DOP80 diet (2.94 compared to 3.02 for the other diets) and by the lactation phase (*p* < 0.001), with the greatest value being observed for late lactation (3.06 compared with 2.92 for mid-lactation and 3.00 for early lactation) (Table 3).

## 4. Discussion

### 4.1. Phenolic Compounds, Fat-Soluble Vitamins and Antioxidant Capacity

Antioxidants from DOP in the current and previous study [21] were transferred from the feed to the milk, in accordance with data collected by Santos et al. [18] for cows fed citrus pulp. The retinol and α-tocopherol contents of milk varied depending on factors such as herbage intake, the botanical and vegetative stage of the pasture, and the supply of feed concentrate in diets [28,35]. Although individual intake was not measured in the present study, a lower intake of concentrate per animal was indirectly achieved by reducing each group’s concentrate consumption towards the end of lactation while maintaining a similar forage intake throughout the complete lactation, which could explain the increase in fat-soluble vitamins between early and late lactation, especially retinol. Moreover, citrus pulp notoriously contains phenolic molecules with antioxidant properties [19], and it has been shown that citrus waste is richer in polyphenols than the citrus consumed by humans [36]. Therefore, the lower intake of concentrate–and, consequently, of orange pulp pellets–towards the end of the lactation period could explain the decrease in TPC and TAC between early and late lactation.

According to Luciano et al. [19], among lambs fed diets supplemented with dried citrus pulp (DCP), the α-tocopherol played a major role in improving the antioxidant capacity of the lamb’s muscle. Indeed, the present study observed a high correlation between TAC, using the ABTS method, and the TPC content of the goat’s milk; however, no significant correlation was found between TAC and α-tocopherol, potentially because the method monitors the antioxidant activity of both whey and total milk and is more sensitive to caseins and other low-molecular-weight compounds [37,38]. Further research should undoubtedly clarify DOP’s antioxidant effect on the milk of ruminants.

### 4.2. Fatty Acid Composition

This is the first report on long-term DOP replacement in the diet of dairy goats that provides detailed information on the FA profile of the resultant milk fat. Additionally, information on the FA profiles of milk from local goat breeds is scarce, with more studies available considering cosmopolitan breeds, likely because of their economic impact [39]. Among the main factors, diet appeared to be most important for milk’s FA composition [15]. Although the effect of the lactation stage on the FA profile of goat’s milk has been described by several authors, the pattern previously described for FA throughout lactation is controversial [39]. Most studies have found that it is difficult to separate the effect of the physiological changes produced by lactation from the effect of the feeding regimen, which also changes during the different stages.

According to previous findings, the main FAs in milk fat are C16, C18:1c9, C18, C10, and C14 (showing content above 7–8% of total FAs), comprising about 75% of total FAs [40]; this is true for both Payoya goats [27,28] and other native breeds [39,41]. The present study observed significant effects of the main factors and the interactions between them for most individual FAs, FA groups, and FA indices. Regarding diet, a study by Ibáñez et al. [20], in which total barley grain was replaced by DCP in mid-lactation Murciano-Granadina goats, showed no effect on FAs with 4 to 15 carbon atoms but significant differences for other FAs. Regarding the lactation stage, and similarly to this study’s results, Strzalkowska et al. [41], who considered the milk of Polish White Improved goats on days 60, 120, and 200 (approximate peak of lactation, mid, and late lactation), observed lactation stage to have no effect on the proportion of C4:0, C14:0, C16:0, C17:0 or MCFA. However, in studies on the milk of Italian goat breeds (from 4 to 24 weeks of lactation) [39], the Brown Shorthair goat breed (from 9 to 37 weeks of lactation) [42] and Murciano-Granadina goat breed (during the full 190-day lactation period, work with a similar experimental design to our study) [43], lactation phase affected all or almost all FAs and indices. Other differences observed between the results of the present research and those of previous studies were mainly related to the different feeding regimens used in each study.

The FAs with 16 or fewer carbon atoms are derived from de novo synthesis, whereas those with 18 or more come from the diet or from lipid mobilization [44]. Especially in late lactation, the inclusion of DOP in the diet resulted in a reduction in many of the FAs synthesized de novo by the mammary gland, such as total SCFA, C6, C8, C12, and C16:1. This could be due to the greater content of C18:1 FAs in DOP diets during late lactation—especially in the DOP80 group—which have an important inhibitory effect on the de novo synthesis of FAs [45]. Furthermore, for the DOP80 group, the higher C18:0 and C18:1 FA content produced during early and mid-lactation might explain the lower C16:0 content (see Table 4). The higher C16:1 content found in the milk of the CD goats during late lactation might be due to the greater desaturation activity of C16:0 in the mammary gland (see Table 4). Similarly to this study, Ibáñez et al. [20] found no effect on FAs with 4 to 15 carbon atoms during mid-lactation. Regarding lactation stage, greater C4:0 to C12:0 FA content was observed in the early rather than the late stage, which could be related to the greater supply of concentrate during the early stage, which might be responsible for increased de novo FA synthesis [46]. This is in spite of the high levels of C18:0 and C18:1 LCFA content observed during this phase, likely due to the mobilization of LCFA from adipose tissues as a consequence of the negative energy balance that normally occurs after parturition (see Table 4). Additionally, the higher levels of SCFA and MCFA content in mid-lactation could be a consequence of higher administration of supplementary concentrate, while the lower percentages of C18:0 and C18:1 are likely due to less mobilization of LCFA from adipose tissues as a result of a positive energy balance (see Table 4). The content of palmitic acid—the predominant FA in milk fat—was particularly high during late lactation; consequently, total MCFA was also substantial, which follows the similar results of other studies [28,39] and could also be related to positive energy balance.

During late lactation, the CD diet produced higher values for C15 and elaidic acid (C18:1 n-9 *trans*) than the DOP diets, which follows the observations of Ibañez et al. [20]. According to some authors [47], the starch content in the feed ration substantially contributes to a high elaidic acid percentage. The odd FAs (C15:0 and C17:0) are considered potential biomarkers of rumen activity and might be partially synthesized endogenously from rumen substrates in the mammary gland [47,48]. Additionally, an increased percentage of amylolytic bacteria possibly increases anteiso and linear forms of odd FA in milk, probably as a consequence of stressful conditions (e.g., a decrease in rumen pH near the acidosis) [47], suggesting a negative long-term impact of CD on rumen’s bacterial metabolism and fermentative activity. Besides, odd FA contents were affected by goat breed [39], with the major content of those FAs in native goat’s milk (as the goat breed of the present study)—as compared to that derived from the cosmopolitan Saanen breed—potentially related to a low level of adaptability to the predominance of concentrate in the feed ration. However, in the study of Ibáñez et al. [20], the lower content of C15:0 in the milk of DOP goats and the ammonia-N results found in rumen liquid were related to a worse impact of these diet on rumen activity. Thus, further research should clarify the effect of orange pulp throughout lactation on rumen function.

Diet treatment mainly affected the octadecenoic acids of milk fat in late lactation, with lower C18:0 content and higher content of most C18 unsaturated FAs, for DOP diets compared with CD diet (see Table 4). The lower C18:0 content in the milk of animals fed DOP diets might be partially due to the greater C18:0 desaturation activity in the mammary gland, a consequence of the balanced maintenance between saturated and unsaturated FAs for milk fluidity control [49]. Although the apparent transfer of C18 unsaturated FAs to milk is affected by several factors, the major determinant is the extent of its biohydrogenation in the rumen [50]. Accordingly, Santos-Silva et al. [51] evaluated ewe milk during early lactation, including the effect of substituting cereals with DCP in rations supplemented with 5% soybean oil, observing an incomplete biohydrogenation pattern in the citrus diet. In the present study, the goat intake of C18 unsaturated FAs was probably lower in the DOP diets than the CD diet (see Table 1); thus, the effect of replacing cereals with DCP on milk biohydrogenation intermediary output could also be partially explained by the more incomplete biohydrogenation pattern. Additionally, citrus by-products are rich in plant secondary compounds, such as phenolic compounds, as has been detected by this and other studies [18,19,52]. This has been reported to disturb rumen biohydrogenation pathways and promote the accumulation of biohydrogenation intermediaries, presumably by inhibiting the last reductive step [53]. The participation of these compounds in the modulation of rumen biohydrogenation pathways could also explain this study’s results. Indeed, Santos et al. [18] replaced barley with DCP in the diet of lambs and also found a reduction of C18:0 and an accumulation of biohydrogenation intermediate concentrations in the blood plasma.

Regarding human health, the most favorable MUFA/SFA and PUFA/SFA ratios were observed in milk derived from DOP diets, especially during the late stage. The reduced total SFA content—in particular of C18:0—and the increased MUFA content in milk from goats fed the DOP80 diet—in comparison with the CD diet (and considering the intermediate values for DOP40)—improved those ratios (Table 4). Regarding n-3 and n-6, DOP diets showed greater values than the CD diet for the indispensables α -C18:3 n-3 and C18:2 n-6 *cis* during late lactation. Total n-3 was only affected by lactation stage, with the higher content levels in the late stage—when the forage-to-concentrate ratio was higher—possibly due to the greater intake of forage than in early and mid-stages. The results obtained for the n-6/n-3 index for all diet treatments (ranging from 9.4 to 11.8) were higher than those recommended to prevent CVD. Nonetheless, the least content was observed in the milk derived from CD goats during late lactation, a consequence of the lower n-6 content of the diet. The CLA *cis*-9, *trans*-11 presented small differences between the diets only during late lactation, with the higher content in the milk of CD goats possibly due to a greater CLA index in the mammary gland (see Table 4). In general, milk richer in unsaturated FAs produces lower AI and TI indices, indicating that the milk might be healthier [5]. In this study, while AI was only affected by the lactation stage, TI in the milk from DOP80 goats was lower than that observed for the other diets (Table 3), likely related to the lower C18:0 content and higher MUFA content levels in milk derived from the DOP80 goats. The present study’s results are similar to those obtained by Ibáñez et al. [20] during mid-lactation; however, that study only included MCFA, SFA, MUFA, PUFA, and AI index values, and only detected differences in PUFA (with higher content levels being detected in the control diet compared to the DOP diet).

## 5. Conclusions

This study found that most FA and antioxidant parameters for goat’s milk were affected by diet (where 40% or 80% of cereals were replaced with DOP over a complete 180-day lactation), stage of lactation, and interaction between both main factors. The levels of α-tocopherol, TPC, and TAC in milk increased as the percentage of DOP replacing cereals increased. Regarding the FA profile, including DOP in goat diets might improve the nutritional indices of FAs in milk for human health, especially during late lactation, because it contributes to the reduction of the thrombogenicity index and increases the MUFA/SFA and PUFA/SFA indices, as well as the indispensables α-C18:3 n-3 and C18:2 n-6 *cis*. Although observation of this effect throughout lactation could suggest the positive long-term impact of DOP on rumen microbial populations and fermentative activity, further research should undoubtedly clarify DOP’s effect on rumen function. Additionally, differences throughout lactation should be linked with physiological and feeding regimen changes, such as the mobilization of FA from adipose tissues in early-to-mid lactation and the increase in forage intake towards the end of lactation. In conclusion, replacing between 40% and 80% of cereals with DOP in the diet of dairy goats over the course of a complete 180-day lactation represents a plausible alternative use for residues derived from the agro-industrial sector, with human health potentially benefitting from increasing the healthy FA profile and antioxidant capacity of milk.

## Figures and Tables

**Table 1:** Ration ingredients and proximate and fatty acid (FA) composition of the experimental diets throughout the lactation period.

Items	Early- and Mid-Lactation Experimental Diets ^1^			Late-Lactation Experimental Diets ^1^		
	Control	DOP40	DOP80	Control	DOP40	DOP80
Ration ingredients, % DM basis						
Alfalfa hay	17.4	17.5	17.6	20.2	20.3	20.4
Concentrate						
Dehydrated orange pulp (pellets)	0	20.0	40.0	0.00	19.4	38.6
Grain oats	22.1	13.2	4.38	21.4	12.8	4.24
Grain barley	8.53	5.11	1.70	8.28	4.96	1.65
Grain corn	19.3	11.6	3.89	18.8	11.3	3.77
Soy flour, 44%	7.31	10.2	13.0	7.09	9.92	12.6
Sunflower pellets, 28%	12.8	12.5	13.8	12.5	12.1	13.5
Grain peas	10.3	8.12	4.05	10.0	7.87	3.93
Salt	0.41	0.41	0.41	0.39	0.39	0.39
Stabilized lard	0.41	0.00	0.00	0.39	0.00	0.00
Vitamins and minerals	1.31	1.31	1.32	1.01	1.01	1.02
Proximate composition and nutritive value, % DM						
DM, %	87.2	87.6	88.1	87.1	87.1	88.1
Crude protein	17.8	16.6	18.4	20.9	18.7	18.3
Neutral detergent fiber	24.3	22.6	25.4	29.8	26.6	28.3
Acid detergent fiber	12.3	15.9	19.0	14.7	15.2	16.8
Acid detergent lignin	4.14	4.19	5.45	3.09	3.13	3.43
Ether extract	3.42	2.31	1.71	2.63	1.85	1.43
Ash	4.82	6.44	7.50	6.50	7.47	8.64
Gross energy, kcal/g DM	4.56	4.44	4.42	4.37	4.31	4.25
Forage unit for lactation, UFL/kg	0.98	0.98	0.97	0.98	0.98	0.96
Protein digestible in the intestine (PDI)	10.5	10.9	11.3	10.4	10.4	11.4
Total phenolic compounds, g gallic acid equivalents/kg DM	5.29	6.74	8.57	5.68	7.07	8.67
Total antioxidant capacity, mmol trolox equivalents/kg DM	14.9	20.4	26.3	11.4	19.4	31.5
α-tocopherol, mg/kg DM	3.18	26.5	70.1	-	-	-
FA composition, % total FA						
C8:0–C14:0	1.10	2.99	4.58	3.88	4.22	6.11
C16:0	23.7	23.6	27.7	23.3	25.1	27.6
C16:1	0.77	0.62	0.64	0.57	0.42	0.69
C18:0	9.02	9.83	11.4	8.90	8.98	11.9
C18:1 n-9 *cis*	26.0	23.6	20.6	26.9	20.7	15.5
C18:2 n-6 *cis*	37.0	35.3	30.2	34.2	36.3	31.7
C18:3 n-6	0.14	0.19	0.47	0.29	0.20	0.38
C18:3 n-3	2.22	3.85	4.38	1.93	4.00	6.08
∑SFA	33.8	36.5	43.7	36.1	38.3	45.6
∑MUFA	26.8	24.2	21.2	27.5	21.1	16.2
∑PUFA	39.4	39.3	35.1	36.4	40.5	38.2
∑n-6	37.2	35.5	30.7	34.5	36.5	32.1
∑n-3	2.22	3.85	4.38	1.93	4.00	6.08
n6/n3	16.7	9.22	7.01	17.8	9.13	5.28

^1^ The control group’s diet was based on a commercial concentrate, with alfalfa hay as forage; for the DOP40 and DOP80 groups, 40% and 80% of the concentrate’s cereal was replaced with DOP.

**Table 2:** Effects of experimental diets ^1^ and lactation phase on resultant milk’s fat-soluble vitamins, phenolic compounds, and antioxidant capacity.

Item	Diet (D) ^1^			Lactation Phase (LP))			SEM	*p* ^2^		
	Control	DOP40	DOP80	Early	Mid	Late		D	LP	D × LP
Fat, %	4.11	4.19	3.85	3.72c	3.96b	4.52a	0.07	ns	***	ns
Fat yield, g/day	60.7	57.4	56.6	66.5a	54.7b	52.6b	2.16	ns	**	ns
Retinol, μg/100 g	4.84	6.64	6.07	3.09c	4.59b	9.11a	0.94	ns	**	ns
α-Tocopherol, μg/100 g	27.1b	28.2b	45.2a	35.6a	25.1b	38.8a	2.07	**	**	ns
Total phenolic compounds, mg gallic acid equivalents/L	46.3c	69.1b	97.0a	83.3a	75.7b	56.1c	2.73	***	***	***
Total antioxidant capacity, μmol trolox equivalents/mL	6..44c	9.39b	12.0a	10.1a	9.06b	8.61c	0.27	***	***	*

Means with different letters (a, b, c) within each row differ significantly (*p* ≤ 0.05); ^1^ See Table 1; ^2^ ns, not significant (*p* > 0.05); *, *p* ≤ 0.05; **, *p* < 0.01; ***, *p* < 0.001.

**Table 3:** Effects of experimental diets ^1^ and lactation phase on fatty acid (FA) composition of milk.

Item ^3^ (g/100 g FA)	Diet (D) ^1^			Lactation Phase (LP))			SEM	*p* ^2^		
	Control	DOP40	DOP80	Early	Mid	Late		D	LP	D × LP
C4:0	3.65	3.48	3.57	3.60a	3.74a	3.34b	0.03	ns	***	ns
C6:0	4.85a	4.52b	4.67ab	4.78a	4.84a	4.39b	0.04	**	***	**
C8:0	4.17a	3.87b	4.00b	4.10a	4.15a	3.76b	0.03	***	***	*
C10:0	9.86a,b	9.53b	10.13a	9.69b	10.62a	9.14c	0.08	**	***	ns
C11:0	0.07a	0.06b	0.06b	0.07a	0.07a	0.06b	0.00	***	***	ns
C12:0	5.58a	5.40b	5.39b	5.46a	5.59a	5.29b	0.04	*	**	ns
C13:0	0.06	0.06	0.06	0.06a	0.06a	0.05b	0.00	ns	***	ns
C14:0	7.30b	7.46a,b	7.54a	7.09b	6.99b	8.22a	0.06	*	***	***
C14:1	0.19	0.19	0.19	0.19	0.19	0.19	0.00	ns	ns	ns
C15:0	0.52	0.51	0.51	0.52	0.52	0.49	0.01	ns	ns	***
C15:1	0.03	0.03	0.03	0.03	0.03	0.03	0.00	ns	ns	ns
C16:0	24.4b	25.1a	24.4b	24.6b	24.2c	25.3a	0.10	**	***	*
C16:1	1.01	1.01	1.00	1.01	1.01	1.00	0.01	ns	ns	**
C17:0	0.33	0.35	0.32	0.32	0.32	0.35	0.00	ns	ns	ns
C17:1	0.09b	0.11a	0.10a,b	0.09b	0.09b	0.12a	0.00	**	***	***
C18:0	11.4	11.3	10.9	11.5a	10.8b	11.2a,b	0.10	ns	**	***
C18:1 n-9 *trans*	0.97a	0.91b	0.89b	0.94a	0.96a	0.85b	0.01	***	***	**
C18:1 n-11 *trans* (VA)	0.68	0.70	0.67	0.67	0.68	0.71	0.01	ns	ns	**
C18:1 n-9 *cis*	19.6b	20.0a,b	20.3a	19.9a,b	19.7b	20.3a	0.09	*	*	*
C18:2 n-6 *trans*	0.15b	0.19a	0.17a	0.14b	0.15b	0.23a	0.01	**	***	***
C18:2 n-6 *cis*	3.20	3.30	3.23	3.31a	3.35a	3.08b	0.03	ns	***	***
γ -C18:3 n-6	0.11a	0.10b	0.09b	0.09b	0.09b	0.12a	0.00	***	***	***
α -C18:3 n-3	0.21	0.22	0.22	0.21b	0.21b	0.23a	0.00	ns	*	**
CLA *cis*-9, *trans*-11 (RA)	0.69a	0.67a,b	0.65b	0.68a,b	0.69a	0.64b	0.01	*	*	**
CLA *trans*-10, *cis*-12	0.01	0.02	0.02	0.01b	0.01b	0.02a	0.00	ns	***	***
C20:0	0.22a	0.20b	0.20b	0.21a	0.21a	0.19b	0.00	*	*	ns
C20:1 n-9	0.04	0.04	0.04	0.04	0.04	0.04	0.00	ns	ns	ns
C20:2	0.06	0.06	0.06	0.06	0.06	0.06	0.00	ns	ns	ns
C20:3 n-3	0.02	0.03	0.02	0.03	0.03	0.03	0.00	ns	ns	ns
C20:3 n-6	0.03	0.03	0.03	0.03	0.03	0.03	0.00	ns	ns	***
C20:4 n-6	0.20	0.20	0.20	0.19b	0.20b	0.21a	0.00	ns	**	***
C20:5 n-3	0.03	0.03	0.03	0.03	0.03	0.03	0.00	ns	ns	ns
C21:0	0.02	0.02	0.02	0.02	0.02	0.02	0.00	ns	ns	ns
C22:0	0.11	0.11	0.10	0.12a	0.12a	0.09b	0.00	ns	***	ns
C22:1 n-9	0.02	0.02	0.02	0.02	0.02	0.02	0.00	ns	ns	ns
C22:2	0.01	0.01	0.01	0.01b	0.01b	0.02a	0.00	ns	***	ns
C22:5 n-3	0.05	0.05	0.05	0.05	0.06	0.05	0.00	ns	ns	***
C22:6 n-3	0.02	0.02	0.02	0.02	0.02	0.02	0.00	ns	ns	ns
C23:0	0.02	0.02	0.02	0.02	0.02	0.02	0.00	ns	ns	ns
C24:0	0.02	0.02	0.02	0.02a	0.02a	0.01b	0.00	ns	*	ns
C24:1	0.01	0.01	0.01	0.01	0.01	0.01	0.00	ns	ns	ns
SFA	72.5a	72.0a,b	72.0b	72.2	72.3	72.0	0.09	*	ns	***
MUFA	22.6b	23.0a,b	23.2a	23.0a,b	22.7b	23.3a	0.08	*	*	*
PUFA	4.82	4.94	4.81	4.87a,b	4.94a	4.77b	0.03	ns	*	***
SCFA	22.5a	21.4b	22.4a	22.2b	23.3a	20.6c	0.14	***	***	**
MCFA	39.6b	40.3a	39.6b	39.4b	39.1c	41.1a	0.13	**	***	**
LCFA	37.9	38.3	38.0	38.4a	37.6b	38.3a	0.11	ns	**	***
n-3	0.34	0.34	0.34	0.33b	0.35a,b	0.36a	0.00	ns	***	ns
n-6	3.69	3.83	3.72	3.77a,b	3.82a	3.67b	0.03	ns	*	***
CLA total	0.71a	0.69a,b	0.67b	0.69a,b	0.70a	0.66b	0.01	*	*	***
n-6:n-3	10.8	11.2	10.9	11.5a	11.0b	10.4c	0.11	ns	***	**
MUFA/SFA	0.31b	0.32a,b	0.33a	0.32	0.31	0.32	0.00	*	ns	**
PUFA/SFA	0.07	0.07	0.07	0.07	0.07	0.07	0.00	ns	ns	***
DI C16:0	0.04	0.04	0.04	0.04	0.04	0.04	0.00	ns	ns	**
DI C18:0	0.65b	0.66a,b	0.67a	0.65	0.66	0.66	0.00	**	ns	***
CLA index	0.50	0.49	0.49	0.50a	0.50a	0.48b	0.00	ns	*	***
AI	2.16	2.16	2.14	2.10b	2.10b	2.27a	0.01	ns	***	ns
TI	3.02a	3.02a	2.94b	3.00b	2.92c	3.06a	0.02	*	***	ns

For each main effect type, means with different letters (a, b, c) within each row differ significantly (*p* ≤ 0.05); ^1^ See Table 1; ^2^ ns, not significant (*p* > 0.05); *, *p* ≤ 0.05; **, *p* < 0.01; ***, *p* < 0.001; ^3^ SCFA, short-chain FAs (C4:0-C10:0); MCFA, medium-chain FAs (C11:0-C17:1); LCFA, long-chain FAs (C18:0-C24:1); DI C16:0, desaturation index of C16:0 [C16:1 FAs/(C16:0 + C16:1 FAs)]; DI C18:0, desaturation index of C18:0 [C18:1 FAs/(C18:0 + C18:1 FAs)]; CLA desaturase index [rumenic acid, RA/(vaccenic acid, VA + RA)]; AI, atherogenic index [(C12:0 + 4 x C14:0 + C16:0)/(MUFA + PUFA)]; TI, thrombogenic index [(C14:0 + C16:0 + C18:0)/(0.5 x MUFA + 0.5 x n-6 PUFA + 3 x n-3 PUFA) + (n-3 PUFA/n-6 PUFA)].

**Table 4:** Effect of interaction between dietary treatment ^1^ and lactation phase (early, mid, late) on the fatty acid (FA) composition of milk.

Item (g/100 g FA)	Early			Mid			Late		
	Control	DOP40	DOP80	Control	DOP40	DOP80	Control	DOP40	DOP80
C6:0	4.97a	4.57b,c	4.84a,b	4.90a,b	4.88a,b	4.72a,b,c	4.70a,b,c	4.10d	4.45c,d
C8:0	4.28a	3.91b,c	4.15a,b	4.22a,b	4.19a,b	4.04a,b,c	4.02a,b,c	3.52d	3.82c,d
C14:0	7.15c	7.10c	7.01c	7.01c	7.06c	6.89c	7.72b	8.18b	8.71a
C15:0	0.49a.b	0.56a	0.50a,b	0.52a,b	0.51a,b	0.54a	0.55a	0.45b	0.49a,b
C16:0	24.3b,c	25.2a,b	24.0c	24.3b,c	24.6a,b,c	23.6c	24.5a,b,c	25.5a	25.6a
C16:1	0.95b	1.09a	0.98b	1.00a,b	0.99a.b	1.04a.b	1.08a	0.96b	0.97b
C17:1	0.09c	0.08c	0.09c	0.09c	0.09c	0.09c	0.09c	0.15a	0.12b
C18:0	11.0b,c,d	11.9a,b	11.6a,b,c	10.7c,d	10.6c,d	11.2b,c	12.4a	11.4a,b,c	10.0d
C18:1 n-9 *trans*	0.97a	0.90a,b	0.96a	0.98a	0.97a	0.93a	0.95a	0.84b,c	0.78c
C18:1 n-11 *trans*	0.70a,b	0.64b	0.66b	0.69a,b	0.69a,b	0.66b	0.66b	0.76a	0.69a,b
C18:1 n-9 *cis*	19.8a,b,c	20.0a,b,c	20.0a,b,c	19.4b,c	19.3c	20.3a,b,c	19.5b,c	20.6a	20.5a,b
C18:2 n-6 *trans*	0.15b	0.14b	0.15b	0.15b	0.15b	0.14b	0.15b	0.28a	0.23a
C18:2 n-6 *cis*	3.45a	3.16a,b	3.35a,b	3.41a,b	3.38a,b	3.26a,b	2.73c	3.36a,b	3.07b,c
γ -C18:3 n-6	0.08b,c	0.09b,c	0.08b,c	0.09b,c	0.09b,c	0.09b,c	0.16a	0.10b	0.11b,c
α -C18:3 n-3	0.22a,b,c	0.20c	0.21b,c	0.22a,b,c	0.21b,c	0.21b,c	0.21b,c	0.24a	0.23a,b
CLA *cis*-9, *trans*-11	0.65b,c	0.71a,b	0.68a,b	0.68a,b	0.68a,b	0.71a,b	0.74a	0.63b,c	0.57c
CLA *trans*-10, *cis*-12	0.01b	0.01b	0.01b	0.01b	0.01b	0.01b	0.01b	0.02a	0.02a
C20:3 n-6	0.03b	0.04a	0.03b	0.03b	0.03b	0.04a	0.04a	0.03b	0.03b
C20:4 n-6 (ARA)	0.20b	0.18b	0.20b	0.20b	0.20b	0.19b	0.19b	0.22a	0.20b
C22:5 n-3 (DPA)	0.04b	0.05a,b	0.04b	0.06a	0.06a	0.06a	0.06a	0.04b	0.05a,b
n-6	3.92a,b	3.61b,c	3.81a,b	3.88a,b	3.86a,b	3.72a,b	3.28c	4.01a	3.64a,b,c
CLA total	0.66b,c	0.72a,b	0.69a,b	0.70a,b	0.69a,b	0.72a,b	0.76a	0.65b,c	0.59c
n-6:n-3	11.8a	11.2a,b	11.6a	11.1a,b	11.1a,b	10.8a,b	9.41c	11.3a,b	10.2b,c
MUFA/SFA	0.32a,b	0.32a,b	0.32a,b	0.31b	0.31b	0.32a,b	0.31b	0.33a	0.33a
PUFA/SFA	0.07a	0.07a	0.07a	0.07a	0.07a	0.07a	0.06b	0.07a	0.07a
DI C16:0	0.04b	0.04b	0.04b	0.04b	0.04b	0.04b	0.05a	0.04b	0.04b
DI C18:0	0.66a,b	0.64b,c	0.65b,c	0.66a,b	0.66a,b	0.66a,b	0.63c	0.66a,b	0.69a
CLA index	0.48b,c	0.52a,b	0.50a,b	0.50a,b	0.50a,b	0.52a,b	0.53a	0.46c	0.45c

Means with different letters (a, b, c, d) within each row differ significantly (*p* ≤ 0.05).

## Data Availability

The data presented in this study are available on request from the corresponding author.
